# Specific antibodies to recombinant allergens of *Aspergillus fumigatus *in cystic fibrosis patients with ABPA

**DOI:** 10.1186/1476-7961-4-11

**Published:** 2006-07-21

**Authors:** Viswanath P Kurup, Alan P Knutsen, Richard B Moss, Naveen K Bansal

**Affiliations:** 1 Allergy-Immunology Division, Medical College of Wisconsin and Research Service, V A Medical Center, 5000 West National Avenue, Milwaukee, WI 53295, USA; 2 Pediatrics Research Institute, St. Louis University, Health Sciences, 3662 Park Avenue, St. Louis, MO 63110, USA; 3 Division of Pediatric Pulmonology, Stanford University Medical School, 701A Welch Road, Suite 3328, Palo Alto, CA 94304, USA; 4 Marquette University, P.O. Box 1881, Milwaukee, WI 53201, USA

## Abstract

**Background:**

*Aspergillus fumigatus*, a widely distributed fungus, has been implicated in causing life threatening infections as well as severe asthma and allergic diseases in man. Allergic affliction like allergic bronchopulmonary aspergillosis (ABPA) is a disabling lung disease frequently seen in patients with asthma and cystic fibrosis. Immunodiagnosis of the former is comparatively easier due to the availability of purified antigens and sensitive methods. However, this is not true with cystic fibrosis patients where the prevalence of ABPA is fairly high and the morbidity and mortality are significant.

**Methods:**

In the present study, we have evaluated purified recombinant allergens from *A. fumigatus*, namely Asp f 1, f 2, f 3, f 4, and f 6 using ELISA and a semi-automated method (ImmunoCAP). We studied 17 patients each from cystic fibrosis with ABPA, and cystic fibrosis with asthma, 22 cystic fibrosis with no ABPA or asthma, and 11 age matched controls.

**Results:**

The results indicate that no antigen, antibody or method is capable of differentiating cystic fibrosis (CF) with ABPA from other CF patients, although some allergens showed strong reaction or showed more prevalence among the patients studied.

**Conclusion:**

When results of several allergens such as Asp f 1, f 2, f 3, f 4, and f 6 in their binding to IgA, IgG, and IgE antibodies were analyzed, a more strong discrimination of CF patients with ABPA was possible from the other groups studied.

## Background

Allergic bronchopulmonary aspergillosis (ABPA) is a disabling allergic disease frequently seen in patients with asthma and cystic fibrosis [1,2]. The prevalence of ABPA has been estimated to be around 1 to 2% in asthmatics and up to 15% among patients with cystic fibrosis [3]. Over 60 percent of the patients with cystic fibrosis (CF) had atopy and the prevalence of ABPA among those patients have been reported to be over 20% compared to 2% in non-atopic CF patients. Immediate cutaneous reactivity to *A. fumigatus *(Af) antigen was detected in 59% of patients, while enhanced IgG antibodies to Af was detected in 51%, and precipitating antibodies in 42% of ABPA-CF patients. Peripheral blood eosinophilia was evident only in one-third of the patients [4]. The available information thus indicate that *Aspergillus*-specific IgE and IgG are elevated in some patients, while in others no such elevation has been detected, and the values are frequently comparable to CF patients without ABPA. Similarly, total serum IgE and skin tests with Af antigens, antigen-specific histamine release, and pulmonary function tests all have limitations in the diagnosis of ABPA with CF [3,5]. Thus, it is evident that the diagnosis of ABPA-CF is extremely difficult compared to ABPA without CF and the laboratory results are frequently inconclusive. Although a number of Af allergens have been cloned and expressed, most of them have not been evaluated simultaneously for their IgE binding and diagnostic significance. Most of the studies have been carried out using crude Af antigens or, less frequently, with recombinant allergens by ELISA and radioimmunoassays [6-9]. Recently, semi-automatic methods have been introduced using recombinant allergens of *Aspergillus*. In the present study, we employed both ELISA and ImmunoCAP (UniCAP, Pharmacia) utilizing some of the promising recombinant and crude extracts of Af to investigate specific IgE and other antibody isotypes in the sera of different groups of patients with CF and Af-induced allergy and normal controls. The results indicate that no single allergen specificity or antibody isotype specificity can reliably identify ABPA with CF, although majority of such patients showed significantly increased polyclonal response to multiple antigens.

## Materials and methods

### Human sera

Sera from four different groups of subjects were studied for various antibodies to *Aspergillus *allergens. All the 17 ABPA patients were diagnosed according to the criteria recommended by Stevens et al [3]. These include clinical deterioration (cough, wheeze, exercise intolerance, exercise induced asthma, decline of pulmonary function, increased sputum) not attributed to another etiology, total serum IgE over 1000 IU/ml (2400 ng/mL), immediate cutaneous reactivity to *Aspergillus *or *in vitro *presence of serum IgE antibody to Af and either precipitins to Af antigens or abnormal chest radiographs showing infiltrates or mucus-plugging. Included in the study was a set of17 CF patients with asthma and positive epicutaneous test responses to Af antigen but no other criteria of ABPA described above. In addition, 22 subjects with CF but no atopy and 11 sera from apparently normal control subjects were also studied. The institutional human study committees of the respective institutions have approved this research.

### *Aspergillus fumigatus *antigens

#### *Aspergillus fumigatus *extract

The crude culture filtrate extract was made as described before [6,10]. In brief; the fungus was grown in a defined medium for up to 3 weeks in stationary cultures at room temperature. The culture filtrates were separated from mycelial mat and freeze dried after extensive dialysis. The extracts were evaluated for their immunochemical characteristics, and the results were evaluated in comparison with in-house standards. Recombinant allergens Asp f 1, Asp f 2, Asp f 3, Asp f 4, and Asp f 6 were obtained by expressing the cloned genes as described before [6-8]. Expressed proteins were also characterized by immunochemical methods as described before [10-12].

#### Enzyme linked immunosorbent assay (ELISA)

ELISA using Af extracts and recombinant proteins were carried out as described before [6]. In brief, the method used was as follows: The microtiter plates were coated with 5 μg/ml of crude Af extract or the recombinant allergens dissolved in phosphate buffered saline (PBS) pH 7.2 for 1 hour at room temperature. The plates were sealed and kept at 4°C overnight and washed in PBS containing 0.05% Tween-20 (PBS-T). After washing the plate, the wells were blocked with PBS-Tween-20 containing 0.5% bovine serum albumin for 1 hour. This was followed by washing the wells three times with PBS-T. Serum dilutions were then added to each well and the plates incubated at room temperature for 3 hours. The plates were washed as before and mouse anti-human IgE (biotinylated) was added and incubated for 1 hour. After washing, streptavidin peroxidase was added to the wells and the plates incubated again for 1 hour. The enzyme reactivity was developed by adding orthophenylene diamine in citrate buffer. The color development was stopped using 2N H_2_S0_4 _and optical density (OD_490_) measured using an ELISA reader. All the dilutions of sera and reagents and the antigen concentrations used in the solid phase were determined after checkerboard titrations. We studied the specific antibodies belonging to the isotypes of IgE, IgG, IgG_1_, G_2_, G_3_, G_4_, IgA, IgA_1_, and IgA_2 _against crude Af antigens and recombinant Af allergens asp f 1, f 2, f 3, f 4, and f 6.

### ImmunoCAP studies

All sera were also tested for Af specific antibodies using UniCAP 100 assay (CAP; Pharmacia/Upjohn, Kalamazoo, MI). *A. fumigatus *total extract and recombinant Af allergens Asp f 1, f 2, f 3, f 4, and f 6 coupled ImmunoCAPs were used to detect specific IgE in the sera. The procedure followed was exactly according to the protocol recommended by the manufacturer. An ImmunoCAP class of 1 or more (0.35 kU_A_/L) was considered to be positive. Same lot of ImmunoCAPs and reagents were used for all the study.

### Statistical analysis

The mean optical density (OD) values for all the different allergens and antibody types and isotypes were calculated and the results of ELISA and ImmunoCAPs analyzed by multivariate analysis of variance (MANOVA). A 'p' value of 0.05 was considered significant. Post hoc univariate analysis of variance was performed to find significant allergens and antibody isotypes and subtypes [13].

To study the effect of all allergens and antibody isotypes, a discriminant analysis was performed combining all the variables. The discriminant analysis creates a scale or scales based on all the combined variables to allow the maximum possible separation between the groups. This scale(s) is called the discriminant function. The stepwise procedure of the discriminant analysis eliminates the insignificant variables. Deletion of these insignificant variables is needed in order to minimize the estimation errors of the discriminate function. It also provides variables that are not needed in order to achieve the maximum possible separation between groups. Based on the discriminant scores, the various groups such as CF, normal, CF with asthma and CF with ABPA were assessed. The percentage of correct classification was used to measure the strength of the discriminant analysis.

## Results

### Specific IgE antibodies in the sera of subjects

**Figure 1:**
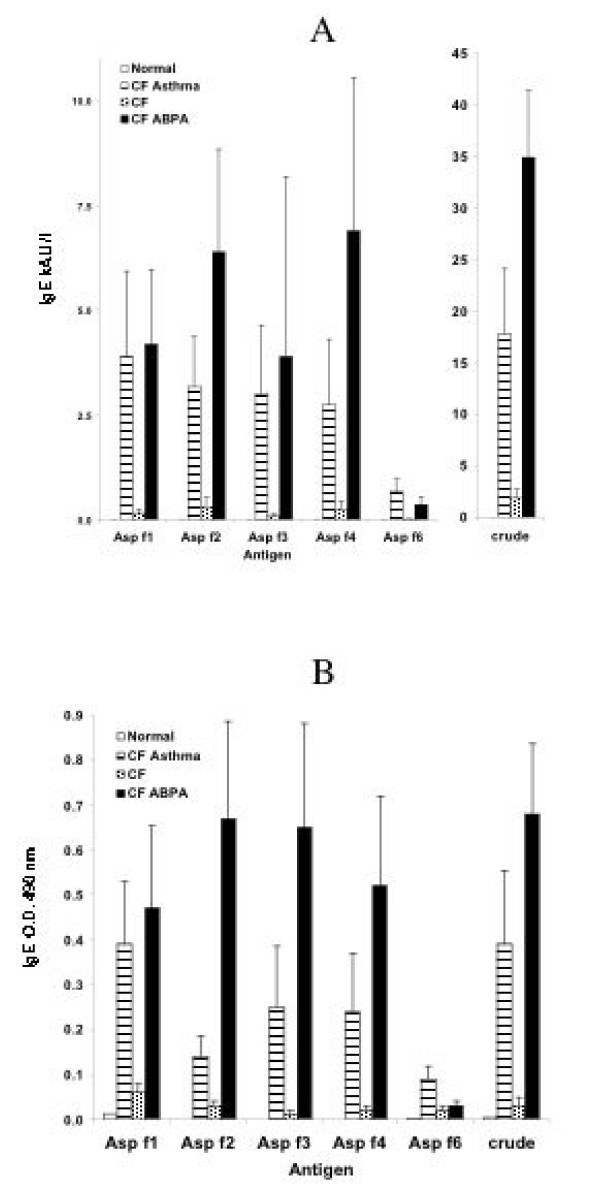
IgE antibodies against various recombinant allergens and crude *Aspergillus *extract. A. IgE measured by ImmunoCAP. B. IgE measured by ELISA.

IgE antibodies against various *Aspergillus *antigens detected by ImmunoCAP and ELISA are shown in Figure 1. Normal subjects and CF patients with no asthma or ABPA failed to demonstrate IgE to any of the recombinant allergens studied. However, the *Aspergillus *extract used in ImmunoCAP reacted with IgE from CF patients with and without ABPA. All five recombinant allergens showed strong reactivity with IgE from CF patients with asthma and CF with ABPA making it difficult to differentiate the different groups. Although, ELISA failed to detect specific IgE in the sera of normal subjects, significant reactivity was noted with CF asthma and CF-ABPA by all tested allergens. Among the various antigens tested, Asp f 2, f 3, f 4, and f 6 showed stronger reactivity with CF-ABPA compared to other groups by both ELISA and ImmunoCap assays. Asp f 6 showed strong reactivity with fewer patients, who failed to show significant reactivity with other antigens. Notably, the IgE antibody levels in the sera of CF-ABPA against both recombinant Af allergens as well as crude Af extract were higher than in CF with asthma, but due to similar reactivity of some patients in both groups, the usefulness of specific IgE for differential diagnosis is questionable as there were no significant differences in the response of specific IgE to *Aspergillus *allergens. None of the normal controls showed any Af specific IgE in their sera against the antigens tested, while CF patients demonstrated only low levels of specific IgE. ImmunoCAP of crude Af antigens showed specific IgE antibody levels in all the tested patients from CF-ABPA and CF asthma.

### Total serum IgE

Although most ABPA-CF patients showed elevated total serum IgE, there was considerable overlap between CF-ABPA and CF-asthma. Occasionally, CF patients also showed elevated total IgE comparable to ABPA-CF.

### *Aspergillus *specific IgG and IgA antibodies

**Figure 2:**
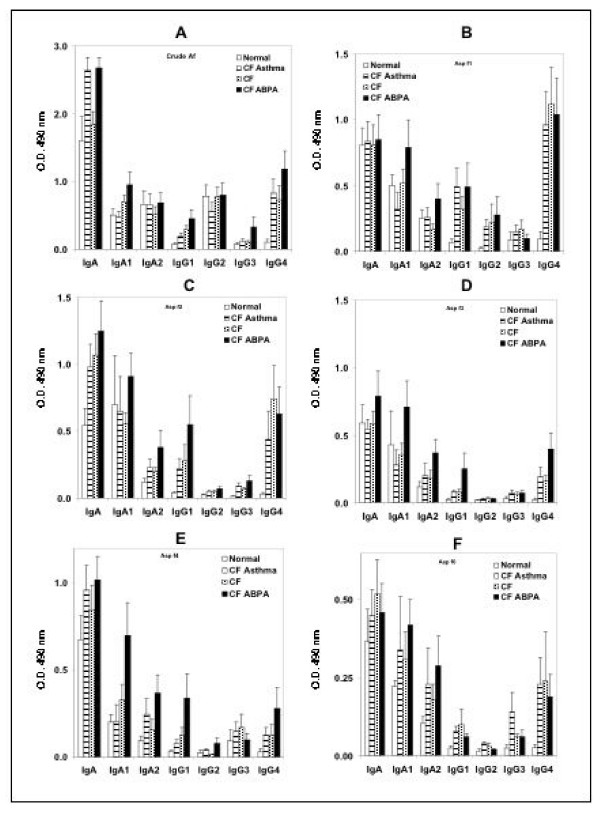
IgA, IgA_1_, IgA_2_, IgG_1_, IgG_2_, IgG_3_, and IgG_4 _antibodies against crude *Aspergillus fumigatus *antigens and various recombinant allergens. A – *A. fumigatus *extract, B – Asp f 1, C – Asp f 2, D – Asp f 3, E – Asp f 4, and F – Asp f 16.

The IgG isotypic responses to the antigens tested showed considerable variation in all groups of patients studied (Figure 2). Only an occasional normal control showed antibody to these allergens when studied by ELISA. Of the 11 normal subjects studied, only one showed IgG isotype response against all the different allergens studied. Two additional normal subjects also showed reactivity to Asp f 4 by ELISA. No significant difference was detected in the IgG_1_, IgG_2_, and IgG_3 _antibodies against the various allergens studied. However, IgG_4 _was consistently elevated in both ABPA CF and CF asthma patients while significant increases were also detected in some patients with uncomplicated CF. Normal controls also showed a specific IgG_1 _responses to crude *Aspergillus *antigen. IgG_4 _antibody levels showed considerable variation among the sera studied from different groups of subjects. Among the IgG isotypes studied, IgG_1 _and IgG_4 _showed higher binding with most of the antigens studied.

IgA antibody responses against different allergens in various groups showed no major differences (Figure 2). Crude *Aspergillus *antigen gave consistently higher values when studied for IgA and IgA subclasses. A number of normal controls also showed strong reactivity with IgA_2_. Although no significant antibody response against any of the different antigens studied was discernible, a marked IgA_1 _response was detected in ABPA-CF patients against most tested recombinant allergens.

### Cannonical-discriminant analysis

**Figure 3:**
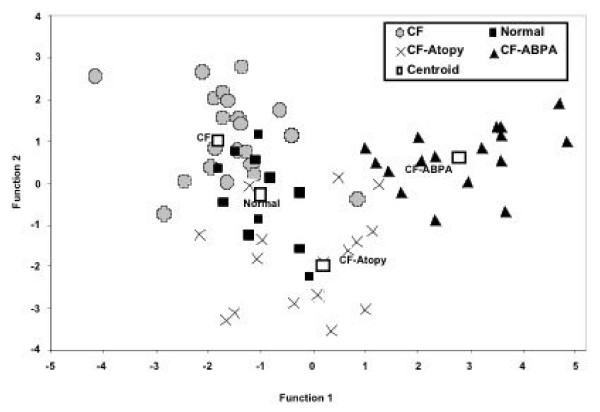
Scatter plot showing discriminant function analysis using IgE, IgA, and IgG antibody responses against Asp f 1, f 2, f 3, f 4, and f 6. Two different functions were analyzed and the results indicate good discrimination of the different groups. ■ normals,  CF, **X **CF with atopy, ▲CF-ABPA patients, and □ the centroids of each group.

The results from different assays were analyzed using discriminant analysis. A preliminary screening of stepwise procedure eliminated IgE and IgG_3 _binding of the serum to Asp f 1, IgG_1_, IgG_3_, and IgG_4 _binding of the sera to Asp f 2, IgG_4 _binding of the sera to Asp f 3, IgG_2_, IgG_3_, and IgG_4 _binding of the serum to Asp f 4 and IgA_2_, IgG_2_, IgG_3_, and IgG_4 _binding of the sera to Asp f 6. IgE, IgA, IgA_1_, and IgG_1 _binding to Asp f 6 was found to be important because eliminating these parameters resulted in significantly large classification errors. The remaining variables when used in the analysis demonstrated significant discrimination between CF asthma, CF-ABPA, CF, and normal groups (Figure 3). The figure 3 displays the first two-discriminant scores (a pair of linear functions of all the inclusive variables) demonstrate significant discrimination between CF asthma and CF-ABPA groups.

To ascertain the power of this analysis, a classification analysis was performed in which patients were classified to one of the four groups (CF asthma, CF-ABPA, CF, and normal) based on the discriminant functions [13]. Overall 95% of the subjects belonging to the various groups were correctly classified. All normal subjects and CF-ABPA patients were correctly classified, while only one out of 22 uncomplicated CF patients, and one out of 17 CF patients with asthma were incorrectly classified using the present system (Table 1).

**Table 1:** Results of the Multiple Function Analysis

	Number of subjects identified in each group based on the analysis				
	
Group	Normal	CF Asthma	CF	CF ABPA	Total
Normal	11 (100%)	0 (0%)	0 (0%)	0 (0%)	11
CF Asthma	1 (6%)	16 (94%)	0 (0%)	0 (0%)	17
CF	0 (0%)	1 (6%)	21 (94%)	0 (0%)	22
CF ABPA	0 (0%)	0 (0%)	0 (0%)	17 (100%)	17

When crude antigen was used in ImmunoCAP or in ELISA, considerable overlap was shown by different groups of patients. However, ELISA identified over 85% of ABPA-CF patients, while no differentiation was possible with IgE ImmunoCAP between CF-ABPA and CF asthma or CF patients.

## Discussion

The diagnosis of CF-ABPA is a more difficult task than diagnosing ABPA without cystic fibrosis [3]. The diagnostic criteria developed to differentiate this disease is also different from those followed in the diagnosis of non-CF-ABPA. Elevated serum total IgE, a common feature considered significant in the diagnosis of ABPA without cystic fibrosis is not as reliable in the differential diagnosis of ABPA with cystic fibrosis [1,12]. Skin test reactivity and the presence of specific IgG are also considered important in the diagnosis of ABPA with and without CF. The multi-functional discriminant analysis using IgE, IgG, IgA and the different recombinant allergens yielded significant results in the differentiation of different patient groups.

It has been shown that crude extract from *A. fumigatus *is a useful reagent for demonstrating specific IgG and IgE from most ABPA patients [14-17]. However, a number of CF patients with and without asthma also demonstrated high levels of both antibodies. Purified recombinant allergens have shown more specificity compared to other allergen preparations when ABPA patients without CF were studied [6,18]. Asp f 1 showed non-specific reactivity with normal controls and CF patients with and without ABPA. However, ABPA patients without cystic fibrosis consistently demonstrate specific antibodies to Asp f 2 compared to other purified recombinant Af allergens [6,9]. In combination with Asp f 3, f 4, and f 6 a number of patients with ABPA demonstrated specific IgE in significant levels. These purified antigens also showed more sensitivity and specificity in comparing patients with ABPA to simple Af allergy.

From the results presented, it can be seen that Asp f 2 demonstrated specific IgE in the sera of CF-ABPA patients, but also showed reactivity with asthmatics and CF patients without any allergy, i.e. high sensitivity, but low specificity. On the other hand, Asp f 3 showed high specificity, but with low sensitivity. Although previous studies have shown that Asp f 3 was significant in differentiating the CF patient groups due to the presence of high levels of *Aspergillus *specific IgE in their sera, we did not find this clear cut difference in the present study [9,16].

In evaluating the ELISA and ImmunoCAP methods, our results indicate that the former was more specific, but less sensitive. The ImmunoCAP of recombinant allergens reacted with more patients and demonstrated Af specific IgE in the sera of ABPA with CF. Similarly a number of asthmatics and CF without allergy also showed high levels of specific IgE to the various antigens included in the ImmunoCaps. ImmunoCAP of crude *Aspergillus *extract demonstrated IgE in over 95% of ABPA-CF, but also reacted with over 78% of asthmatics and 40% of CF patients. Thus, the comparative specificity of ImmunoCap using either crude or purified recombinant allergens of *Aspergillus *is inferior to ELISA, but the latter is also not sufficiently predictive to be acceptable as a reliable diagnostic assay.

The usefulness of specific IgG subclasses in the diagnosis of CF-ABPA has been previously suggested [14]. Although, similar elevations in the levels of IgG_1 _and G_4 _were noted in both CF groups in the present study, these differences were not statistically significant. The discriminant function analysis using all features simultaneously classified most patients in their respective groups. Although all the 17 ABPA-CF patients showed difference in their antibody response, the results on comparison with other groups of patients was found to be significant (Table 1). Generally a discriminant capability of at least 90% is indicated for adapting a test for differential diagnosis (Figure 3).

## Conclusion

The results presented here indicate that the demonstration of specific IgE, IgG, and IgA isotypes with recombinant Af allergens has less usefulness in the diagnosis of CF-ABPA than in non-CF-ABPA. However, in a previous study, Asp f 3 demonstrated slightly enhanced levels of antibody in the sera of patients with ABPA and CF compared to other recombinant allergens [9]. Our results suggest that the evaluation of different antibody isotypes in the sera using a combination of recombinant Af allergens may have added value in the diagnosis of CF with ABPA. The results also suggest that additional relevant antigens and information from cell-mediated immune responses are important in the development of a more definitive diagnosis of ABPA and for the discrimination of ABPA with CF from other CF patients.

## Abbreviations

ABPA – Allergic bronchopulmonary aspergillosis; CF – Cystic fibrosis; ELISA – Enzyme linked immunosorbent assay; Asp f 1 – *Aspergillus fumigatus *allergen; Af – *A. fumigatus*; OD – Optical density; PBS – Phosphate buffered saline; kU_A_/L – Kilounits per liter; MANOVA – Multivariate analysis of variance

## Declaration of competing interests

The author(s) declare that they have no competing interests.

## Authors' contributions

VPK was responsible for the antigen preparation, conducting the various assays, collecting the results and in communicating the results to other collaborators. He has also contributed to the manuscript preparation and in the overall supervision of the project. APK selected the subjects, obtained sera and participated in the planning of the experiments and in the writing of the manuscript. RBM selected the subjects, obtained sera for the study and participated in the planning of the experiments and in the manuscript preparation. NKB actively participated in the study design and was responsible for the statistical analysis of the data. All authors read and approved the final manuscript.
